# Costorage of High Molecular Weight Neurotransmitters in Large Dense Core Vesicles of Mammalian Neurons

**DOI:** 10.3389/fncel.2018.00272

**Published:** 2018-08-21

**Authors:** Adalberto Merighi

**Affiliations:** Laboratory of Neurobiology, Department of Veterinary Sciences, University of Turin, Turin, Italy

**Keywords:** coexistence, co-localization, co-storage, large granular vesicles, neuropeptide, neurotransmission, release, small synaptic vesicles

## Abstract

It is today widely accepted that several types of high molecular weight (MW) neurotransmitters produced by neurons are synthesized at the cell body, selectively stored within large dense core vesicles (LDCVs) and anterogradely transported to terminals where they elicit their biological role(s). Among these molecules there are neuropeptides and neurotrophic factors, the main focus of this perspective article. I here first provide a brief resume of the state of art on neuronal secretion, with primary emphasis on the molecular composition and mechanism(s) of filling and release of LDCVs. Then, I discuss the perspectives and future directions of research in the field as regarding the synthesis and storage of multiple high MW transmitters in LDCVs and the possibility that a selective sorting of LDCVs occurs along different neuronal processes and/or their branches. I also consider the ongoing discussion that diverse types of neurons may contain LDCVs with different sets of integral proteins or dial in a different fashion with LDCVs containing the same cargo. In addition, I provide original data on the size of LDCVs in rat dorsal root ganglion neurons and their central terminals in the spinal cord after immunogold labeling for calcitonin gene-related peptide (CGRP), neuropeptide K, substance P, neurokinin A or somatostatin. These data corroborate the idea that, similarly to endocrine cells, LDCVs undergo a process of maturation which involves a homotypic fusion followed by a reduction in size and condensation of cargo. They also give support to the conjecture that release at terminals occurs by cavicapture, a process of partial fusion of the vesicle with the axolemma, accompanied by depletion of cargo and diminution of size.

## State of Art on High Molecular Weight Neurotransmitter Localization and Function

### Neuronal Secretion and Types of Secretory Vesicles in Neurons

**Figure 1:**
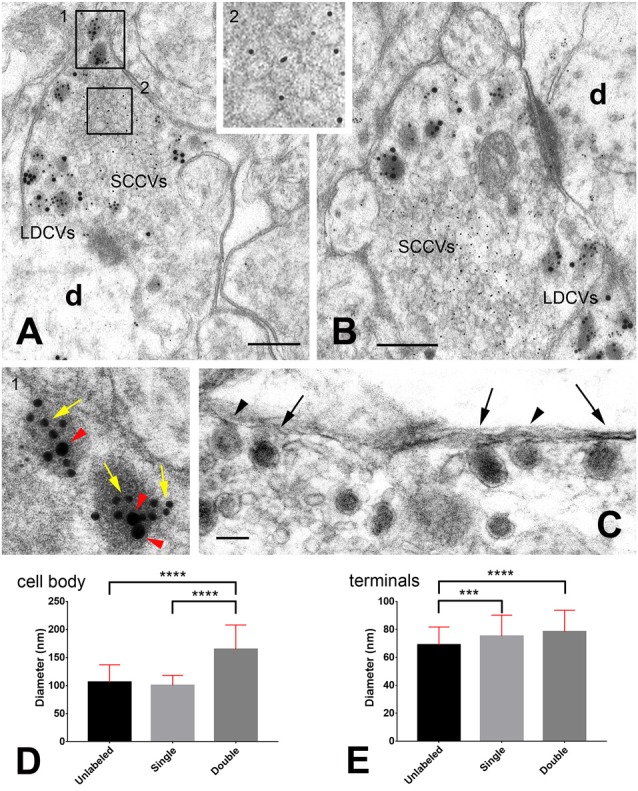
Storage of neuropeptides in rat dorsal root ganglion (DRG) neurons. **(A,B)** Primary afferent terminals in the spinal cord dorsal horn after triple immunogold labeling with antibodies against glutamate (5 nm gold particles), calcitonin gene-related peptide (CGRP; 10 nm gold particles indicated by the yellow arrows in the insert) and substance P (20 nm gold particles indicated by the red arrow heads in the insert). For details on antibodies and labeling methods see Merighi et al. (1991). The two rectangles in **(A)** are shown at higher magnifications in the inserts. **(C)** A particular of an axon terminal in the rat dorsal horn after slice incubation in 75 mM K^+^ to stimulate LDCVs’ exocytosis. Tissue has been processed with the Tannic Acid Ringer Incubation (TARI)-method (Buma et al., 1984). Fixation with tannic acid prior to conventional glutaraldehyde + osmium post-fixation powerfully intensifies the electron density of the substances secreted into the extracellular space. As the cargo of LDCVs is immediately fixed by tannic acid once there, it remains in close apposition to the terminal membrane that appears thicker than after conventional transmission electron microscope (TEM) fixation. Note that three LDCVs in proximity of the axolemma (arrows) display a very electrondense matrix, whereas two other vesicles (arrow heads) display some dissolution of their membranes and are lighter. These images may represent different stages in the process of cavicapture by which neuropeptides may be released at terminals (see also Figure 2). **(D,E)** Diameter (mean ± SD) of unlabeled, single- and double-labeled LDCVs in the neuronal cell body **(D)** and central terminals **(E)** of rat DRG neurons. Note the increase in size of double-labeled LDCVs compared to unlabeled or single-labeled LDCVs in DRGs, likely as a consequence of new cargo addition. Note also that in terminals unlabeled vesicles are smaller than single- and/or double-labeled LDCVs, which, instead, display similar sizes. This observation supports the idea that unlabeled vesicles in terminals may have been depleted of their cargo after cavicapture. Statistics was performed with the GraphPad Prism 7 software. Normality was assessed using the D’Agostino & Pearson normality test. Means were compared using the Kruskal-Wallis non-parametric test followed by Dunn’s multiple comparison. # LDCVs: 346 (cell body), 523 (terminals). ***P = 0.0004; ****P < 0.0001 (two-tailed). Abbreviations: d = dendrite; LDCVs = large dense core vesicles; SSVs = small clear core vesicles. Bars: **(A,B)** = 200 nm; **(C)** = 100 nm.

Differently from other cells, neurons display at least three different types of secretory vesicles, each showing distinctive features as regarding their secretion and biogenesis. Biosynthetic activity in neurons is very intense and, for a substantial part, devoted to synthesize and assembly these vesicles. Such an intensive activity is testified by the abundance of rough endoplasmic reticulum (RER) and the existence of large Golgi complexes in neuronal perikarya. Secretory vesicles are produced along the regulated secretory pathway and store soluble proteins, peptides or low molecular weight (MW) neurotransmitters. Very recent studies have demonstrated that they may also contain small ribonucleic acids (sRNAs), at least in the electric organ of *Torpedo californica* and in mouse synapses (Kim et al., 2015; Li et al., 2015, 2017; Gümürdü et al., 2017). Once assembled, secretory vesicles are actively transported to specific subcellular domains for extracellular delivery in response to appropriate signals. The typical large (75–100 nm) dense core vesicles (LDCVs) in neurons, which mainly concern this perspective article, contain proteins and/or peptides (Figures 1A,B). It may be useful to recall here that LDCVs were originally defined in non-neuronal cell types, where they can be much larger than those found in neurons. This likely is important for understanding the differences between peptide hormone release and neuropeptide release (see below). Proteinaceous molecules contained in neuronal LDCVs are synthesized in the cell body, generally as larger precursors that are commonly referred to as pre-pro-peptides, packaged into LDCVs to be transported to processes and, eventually, delivered into the extracellular space (Merighi, 2017). However, it remains unclear where, along their long journey from cell body to terminals, maturation of LDCVs’ neurotransmitter proteins takes place.

Biochemical studies on undifferentiated PC12 cells, which are devoid of axons, indicate that the maturation of LDCVs is accompanied by sorting non-regulated secretory proteins, including the SNARE proteins, from immature vesicles through the recruitment of clathrin coats, a process that is considered essential for the maturation of LDCVs (see Morvan and Tooze, 2008). In parallel, several observations on the processing of pre-pro-peptides in hypothalamic neurons and their neurohypophyseal axon terminals support that a post-translational cleavage of vasopressin, oxytocin and their neurophysins occurs during axonal transport (e.g., Gainer et al., 1977a,b). These latter studies did not take into consideration the aforementioned modifications of the proteins of the LDCVs’ membrane. However, it was more recently demonstrated that, in cultured trigeminal ganglion neurons, the calcitonin gene-related peptide (CGRP) occurs together with three SNAREs and synaptotagmin in LDCVs and that SNARE proteins (SNAP25, syntaxin one and the synaptobrevin isoforms) were implicated in the exocytosis from LDCVs (Meng et al., 2007). Even more recently, SNAP25, and synaptobrevin isoform 2, as well as SNAP47, were demonstrated to mediate the axonal release of brain-derived neurotrophic factor (BDNF) from cortical neurons (Shimojo et al., 2015). Therefore, it appears that also in *bona fide* neurons SNAREs may be constituent of the mature LDCVs, although it remains to be established whether post-translational axonal cleavage of pre-pro-neurotransmitter proteins is a general rule or rather a peculiarity of the hypothalamic neurosecretory neurons.

In nerve terminals, small (40–50 nm) synaptic vesicles (SSVs) or small clear core vesicles are definitely more numerous than LDCVs and store and deliver small neurotransmitters such as acetylcholine, glutamate (Figures 1A,B), glycine, and gamma amino butyric acid (GABA). Finally, biogenic amines are differently packaged in neuronal processes within the central and peripheral nervous systems (see Hökfelt, 2010). In the latter, biogenic amines are stored in either small (40–60 nm diameter) dense core vesicles (SDCVs) or irregularly shaped LDCVs, depending on the particular population of neurons. Conversely, brain dopamine vesicles do not fall in any of the described classes because they are 70 nm in diameter and clear. At the transmission electron microscope (TEM), SSVs undergoing anterograde axonal transport appear as tubule-vesicular structures of 50 nm diameter and variable length (Tsukita and Ishikawa, 1980). These morphological observations, together with the results of numerous biochemical studies, led to the conclusion that SSVs are locally assembled at synapses and that their protein components are reconstituted as complete synaptic vesicles in the early endosome compartment (Takamori, 2009). Thus, SSVs are characteristically recycled several times and locally filled with neurotransmitter at synapses, for reuse following a series of exocytotic and endocytotic events (Lou, 2018).

Differently from SSVs, LDCVs are depleted after secretion, whereas SDCVs containing noradrenaline are filled with the neurotransmitter during axonal transport, a fact that explains the presence of their characteristic dense core in TEM images (Zhang et al., 2011).

### Assembly, Filling and Release of Neuronal LDCVs

#### Integral Proteins of LDCVs

Independently from their cargo, LDCVs express a series of integral proteins that, biochemically, make them a relatively homogeneous population of vesicles. Among these are the chromogranins (Bartolomucci et al., 2011), which are important regulators of cargo sorting in LDCVs biogenesis, although very recent work in mouse hippocampal neurons has demonstrated that they are not indispensable for LDCVs’ exocytosis (Dominguez et al., 2018).

#### LDCVs’ Cargo

The cargo of LDCVs consists of proteinaceous materials. For the most, these proteins are of small size and currently referred to as neuropeptides (Figures 1A,B). In its original definition, a neuropeptide is a small protein molecule contained in neurons, composed of up to a hundred amino acids. As mentioned, neuropeptides are usually produced as large, inactive precursors, which are then enzymatically cleaved to yield the biologically active peptides. Commonly, precursors contain several molecules of the same neuropeptide and/or more or less structurally related compounds. Storage of neuropeptides and their precursors in LDCVs was first shown in the 80s of the last century with TEM, but, in more recent times, LDCVs were demonstrated to be loaded also with bigger molecules such as BDNF or the glial-derived neurotropic factor (GDNF) and their pro-peptides. There are numerous examples of costorage of multiple peptides within individual LDCVs in various areas of the central and peripheral nervous systems (Merighi, 2017).

**Figure 2:**
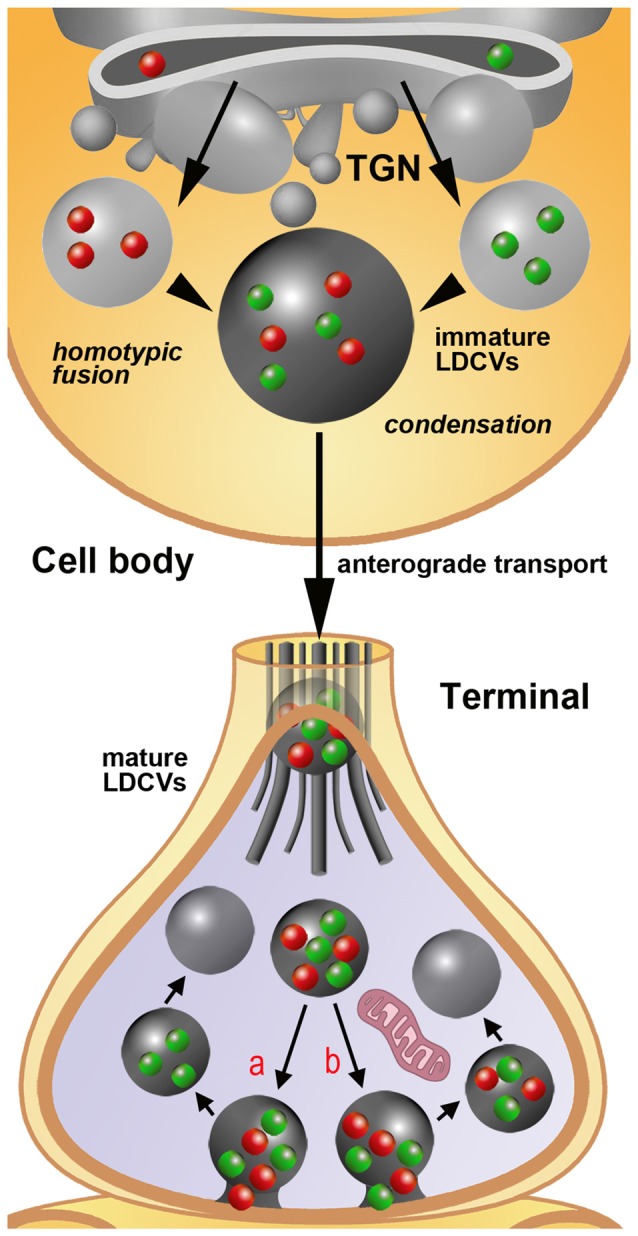
Schematic representation of the process of assembly, filling and release of LDCVs as extrapolated from immunogold staining studies on rat primary sensory neurons in DRGs. For simplicity, only the synthesis of two neuropeptides/proteins (red and green spheres) is depicted. The sizes of immature and mature LDCVs containing no cargo (negative after immunogold labeling), only one of the two molecules (single-labeled) or both molecules (double-labeled) are represented in accordance with the quantitative data reported in Figures 1D,E. In the example, immature LDCVs budding from the trans-golgi network (TGN) contain either one *or* the other neuropeptide and undergo a process of homotypic fusion to give rise to a larger LDCV. This vesicle stores both peptides, but still may be regarded as immature on the basis of its very large size. It subsequently undergoes a process of condensation with a reduction in size that is completed once the axon terminal is reached by anterograde transport. In terminals, LDCVs are smaller and very likely undergo a process of cavicapture to release their cargo (see also the insert #1 of Figures 1A,C). This process may, in theory, yield to a selective: (a) or a non-selective (b) release of the co-stored neuropeptides. Irrespectively of this possibility, individual LDCVs may endure several cycles of cavicapture until they are fully depleted of their cargo.Quantitative analysis (Figure 1E) demonstrates that empty (unlabeled) LDCVs are smaller than those containing only one (and thus single-labeled) of the two co-stored peptides. To make the figure easier, LDCVs are represented without their outer membrane, which is instead clearly visible in tissues subjected to fixation with tannic acid (Figure 1C). For the same reason, SSVs are not rendered in the terminal.

As LDCVs mature, the pro-peptides herein contained undergo proteolysis to become active. This maturation process usually starts in the trans-Golgi network (TGN) and continues in the secretory vesicles themselves (Kögel and Gerdes, 2009). The specific molecular signals that drive the packaging and aggregation of secreted proteins or their characteristic integral membrane proteins into LDCVs within the TGN are starting to be unraveled. Among these, one should recall the WD40 domain protein EIPR-1 and the endosome-associated recycling protein (EARP) complex (Topalidou et al., 2016). LDCVs emerge as immature vesicles from the TGN of the Golgi complex (Figure 2), and it was recently suggested that the conserved coiled-coil protein CCCP-1 intervenes in the homotypic fusion of immature LDCVs during the course of their maturation (Cattin-Ortolá et al., 2017).

#### Release of Cargo From LDCVs

Neuronal LDCVs are not fully equivalent to non-neuronal LDCVs, a point that merits attention in discussing current knowledge on release of cargo from these two classes of LDCVs. Studies of endocrine and exocrine cells with 300–1000 nm LDCVs have often implicated F-actin (and sometimes myosin) in release, because it apparently takes work to move these large vesicles and extrude their contents. In contrast, experiments on synaptic neuropeptide vesicles with cytochalasin and mycalolide B (an F-actin/dynactin disruptor) showed that F-actin does not affect LDCV mobilization, anterograde transport, synaptic capture or evoked synaptic neuropeptide release (Shakiryanova et al., 2005; Cavolo et al., 2015, 2016).

Mechanisms by which LDCV and SDCVs release their cargo include exocytosis, kiss and run or cavicapture (Merighi, 2017). Exocytosis is a relatively slow process that requires complete fusion of the vesicle to the plasma membrane; kiss and run or cavicapture are faster and need the formation of a transient fusion pore (TFP) between the LDCV and the plasma membranes. TFP mechanisms permit a fast transfer of amine neurotransmitters from the inside of SDCVs to the extracellular space. Of these mechanisms, neuropeptide kiss and run was recently demonstrated to occur at nerve terminals in *Drosophila* (Wong et al., 2015) and in mammalian DRG neurons (Wang et al., 2017). It seems also possible that neuropeptides and larger proteins can escape from LDCVs by cavicapture, during which an expansion of the TFP triggers a partial release of large-size neurotransmitters (Figures 1C, 2).

As a rule, individual neurons are capable to produce several high MW transmitters of proteinaceous nature and store them in LDCVs. In mammals, ultrastructural demonstration was provided that e.g., two different neuropeptides and a growth factor with transmitter functions could be co-stored in individual LDCVs (Salio et al., 2007, 2014). Thus, there are at least two main possibilities depicting the modalities of release of these molecules (Figure 2). From one side, all co-stored high MW transmitter could be released together. At opposite, individual molecules could be liberated singularly or in different combinations. A further issue of complexity derives from the very peculiar organization of neurons, which form more or less intricate arborizations of their processes, the latter, in turn, existing in at least two functionally different types, i.e., axons and dendrites.

## Future Directions and Perspectives

Neuropeptides and other proteins that may be synthesized by neurons are known to be physiologically very important, but also play a substantial role in many pathological conditions. However, the biogenesis of LDCVs and the mechanisms governing their molecular composition still remain to be unraveled in full (Hummer et al., 2017).There are, in my opinion, two main lines of research to be pursued in the future for a better understanding of the significance of costorage of high MW transmitters in neurons. The first should primarily investigate the synthesis, storage and maturation of LDCVs, the second their targeting to neuronal processes with an attention to possible differences among specific neuronal populations. Information on both issues is substantially missing, as, at present, investigations have been, for the most, carried out on simple organisms such as *Caenorhabditis elegans* or *Aplysia californica* or performed in mammalian secretory cells other than neurons, such as e.g., the adrenal chromaffin cells. In addition, the relatively small number of investigations in mammalian neurons has basically been carried out on isolated primary cells or cell lines *in vitro*, with few remarkable exceptions discussed below.

### Synthesis and Storage of Multiple High MW Transmitters in LDCVs

Localization of immature LDCVs in neurons remains a difficult task to be performed. It still is unclear which morphological and/or biochemical differences, if any, exist between LDCVs at different stages of maturity. Ultrastructurally, it appears that immature vesicles in chromaffin cells are of heterogeneous size, but all have a dense core. In rat PC 12 cells, the dense core of immature secretory granules (80 nm) is smaller than that of mature granules (114 nm), and on these observation it was hypothesized that one or more immature granules fuse together during maturation (Tooze et al., 1991; Tooze, 1991). Other investigations, again conducted in cells other than neurons, have shown that maturation of LDCVs accompanies with condensation of the matrix and, at least theoretically, a reduction in size that, however, could be compensated by homotypic fusion of immature vesicles stemming from the TGN (Kögel and Gerdes, 2009). Studies in mouse chromaffin cells have shown that the neuronal adaptor protein 3 (AP-3), a vesicle-coat protein that in neurons intervenes in transmitter release, is localized to the TGN and hypothesized that AP-3 is selectively expressed in immature LDCVs (Grabner et al., 2006). More recently, a genome editing study on HID-1 knockout PC12 cells has proposed that the protein, originally demonstrated to be implicated in neuropeptide sorting and secretion in *Caenorhabditis elegans*, influences the early steps in LDCV biogenesis by controlling the formation of their dense core at the TGN (Hummer et al., 2017). Thus vesicle size, expression of neuronal AP-3 and/or HID-1 could be regarded as markers of immature LDCVs in endocrine cells, but this remains to be established in full for neurons.

Pioneering work carried out with the use of multiple immunogold labeling methods more than 25 years ago led to establishing that multiple neuropeptides could be co-stored within individual LDCVs in the cell body and processes of certain primary sensory neurons (for review see Merighi, 2017). More recent work has shown that neuropeptide co-storage also occurs in neurons of the central nervous system (Salio et al., 2007). As neurons normally appear to be producin g more than a single neuropeptide/transmitter protein (Merighi, 2017), it seems highly possible that these proteinaceous molecules are not selectively packaged into different mature LDCVs, but rather form a mix in individual vesicles once they are ready to be transported along axons. I have here analyzed the size of unlabeled, single-labeled and double-labeled LDCVs in the cell bodies of rat dorsal root ganglion (DRG) neurons using different combinations of antibodies against several sensory neuropeptides (CGRP, the tachykinins neuropeptide K, substance P and neurokinin A or somatostatin) with different double immunogold labeling techniques (Merighi and Polak, 1993). It is of interest that in these neurons there is no difference in size between unlabeled and single-labeled LDCVs, whereas double-labeled vesicles are larger (Figure 1D). This observation is consistent with the idea that LDCVs containing just one component of the peptide mix are immature and that the increase in size of mature double-labeled vesicles is a consequence of homotypic fusion with other immature LCDVs containing the second peptide (Figure 2), as shown in endocrine cells (Kögel and Gerdes, 2009). As we have previously demonstrated that mature LDCVs in the central terminals of the DRG neurons may contain a mix of three (and likely even more) neuropeptides/larger proteinaceous transmitters (Salio et al., 2007, 2014), the data herein reported are strongly indicative of the possibility that homotypic fusion may be a general phenomenon through which LDCVs mature before being transported to terminals. Co-stored proteinaceous transmitters occur in LDCVs in remarkably constant ratios. We have e.g., demonstrated that substance P, CGRP and BDNF occur in a stoichiometric ratio of 0.7 BDNF:1 CGRP:1 substance P in neurons of DRGs and central nucleus of amygdala (Salio et al., 2007). Therefore, it seems reasonable that such a ratio is attained at the level of protein synthesis, before individual molecules are directed to the TGN (Figure 2).

The main functional implication of co-storage of bioactive molecules within LDCVs is that the neuropeptides and/or the other high MW transmitter herein contained may be released together and probably act together in determining the response of target cells. It would be interesting to investigate whether or not these co-stored molecules are indeed released in concert, or if some sort of mechanisms would permit a selective release according to functional needs. Under this perspective, the relative rate of individual peptide dissolution from the LDCV core (matrix) might be important, since it is critical for the speed of peptide secretion *in vitro* (for a recent review see Merighi, 2017). I have very recently discussed the possibility that two functionally antagonist subpopulations of peptidergic DRG neurons exchange their information to regulate nociception (Merighi, 2018). These two populations of neurons contained a mix of peptides with either BDNF (Merighi et al., 2008) or GDNF (Salio et al., 2014). It appeared highly probable that the two neurotrophic factors come into play only in particular functional conditions, e.g., when the sensory system was overstimulated under inflammatory conditions. In the future, it would be interesting to check whether these molecules can indeed undergo some sort of selective release, as this would represent an additional regulatory mechanism to finely tune nociception.

### Are LDCVs Selectively Targeted to Different Neuronal Processes?

Whether or not LDCVs are selectively targeted along different neuronal processes and/or their branches remains to be established in full. Our immunogold studies onto the DRG neurons and their central and peripheral projections were indicative of a lack of selectivity, as the same combination of peptides, e.g., substance P and CGRP, is detected at both peripheral (Gulbenkian et al., 1986) and central (Merighi et al., 1991) axonal projections. However, we did not investigate this issue in full, and, to the best of my knowledge, it still stands as an open question.

At the central projections of the peptidergic DRG neurons in spinal cord there is a very large fraction (virtually all?) of the total population of LDCVs containing coexisting neuropeptides/protein transmitters compared to the very limited pool of these vesicles in the cell body of these neurons. Another issue which deserves future investigations is the occurrence and significance of unlabeled LDCVs at axon terminals. I have often interpreted this as a false negative observation, as: *1*. There is a general consensus, also based onto light microscopic and transcriptomics studies, that *all* DRG peptidergic neurons contain their main peptide marker CGRP (Amara et al., 1985). Therefore a lack of CGRP immunoreactivity would be difficult to explain in these neurons; *2*. Antigenicity in post-embedding immunogold labeling techniques on plastic sections (which are surface reactions) may often fall below the limits of sensitivity of these procedures (Merighi et al., 1991). To obtain additional cues on this issue, here I have calculated the size of LDCVs in the central terminals of the rat DRG neurons after CGRP+substance P or CGRP+somatostatin double labeling of the dorsal horn (Figure 1E). Remarkably, and differently from what is observed at the cell body (Figure 1D), double- and single-labeled LDCVs are larger than unlabeled vesicles. These observations give support to the idea that peptides are indeed released from these vesicles through cavicapture (Figure 1C), a process by which individual LDCVs do not disappear after totally flat themselves against the neurolemma as in the well know regular exocytosis of SSVs (Rutter and Tsuboi, 2004)—see also the multi-media annex in Merighi, 2017. If this interpretation is correct, unlabeled LDCVs in the central terminals of the DRG neurons could be regarded as depleted of their cargo and thus not as technical artifacts (Figure 2). This conclusion, as mentioned above, is strongly based on the notion that CGRP is expressed in *all* peptidergic neurons in DRGs. Therefore, to generalize it to different (or all) types of LDCV-containing terminals would require to identify a general peptide marker for each neuronal population, to exclude that unlabeled LDCVs, in terminals other that those investigated here, contain a neuropeptide or other cargo that has simply not been assayed.

It is also of interest that when the mean sizes (±SD) of unlabeled, single- and double-labeled LDCVs are compared between the cell body and central terminals of the DRG neurons, those in perikarya are larger than in terminals for all the three groups (unlabeled LDCVs: cell body 105.8 ± 31.21, terminals 68.91 ± 12.8, *P* < 0.0001; single labeled LDCVs: cell body 100.3 ± 17.93, terminals 75.13 ± 15.09, *P* < 0.0001; double labeled LDCVs: cell body 164.5 ± 43.54, terminals 78.26 ± 15.44, *P* < 0.0001; Mann-Whitney test; #LDCVs = 878). This observation is in accordance with data on endocrine cells where a condensation of LDCVs was demonstrated in parallel with maturation (Kögel and Gerdes, 2009).

### Other Sources of Complexity Still Await to Be Cleared

It is of interest that chromogranin B and phogrin, another integral protein of LDCVs in endocrine cells, have been reported to be specifically contained in LDCVs from excitatory and inhibitory hippocampal neurons, respectively (Ramírez-Franco et al., 2016). This observation deserves further investigations as it opens the yet unforeseen possibility that integral proteins of LDCVs may be different in diverse types of neurons.

Also, a study on the cellular processing of neuropeptide Y (NPY) has demonstrated that differential trafficking of immunoreactive LDCVs occurs in hippocampal vs. hypothalamic neurons (Ramamoorthy et al., 2011). If these observations will be extended to other neuropeptides or proteinaceous cargo of LDCVs, further complexity will be added, as trafficking of neuropeptide-containing LDCVs could be specific for different populations of neurons, and perhaps not determined entirely by the characteristics of the particular peptide *per se*.

## Ethics Statement

The experimental procedures described in this paper were approved by the Ethics Committee of the University of Turin. This study was carried out according to current EU Recommendations on the Care and Use of Experimental Animals.

## Author Contributions

AM conceived and performed the experiments, analyzed data, prepared the figures and wrote the manuscript.

## Conflict of Interest Statement

The author declares that the research was conducted in the absence of any commercial or financial relationships that could be construed as a potential conflict of interest.
